# Epidemiology of human salmonellosis in Czechia ─ a country with the highest European notification rate, 2012 to 2023

**DOI:** 10.2807/1560-7917.ES.2026.31.3.2500223

**Published:** 2026-01-22

**Authors:** Michaela Špačková, Monika Liptáková, Ondřej Daniel, Veronika Vlasáková, Jana Košťálová, Jan Kynčl

**Affiliations:** 1 National Institute of Public Health, Prague, Czechia; 2 Second Faculty of Medicine, Charles University, Prague, Czechia; 3 State Veterinary Administration, Prague, Czechia; 4 Third Faculty of Medicine, Charles University, Prague, Czechia

**Keywords:** Salmonella Infections, Epidemiology, Public Health Surveillance, Gastrointestinal Diseases, Salmonella

## Abstract

**BACKGROUND:**

Salmonellosis is the second most notified food-borne infection in Europe. In Czechia (CZ), the notification rate has consistently been above the European average, five times higher in 2013–2022.

**AIM:**

We aimed to describe the epidemiology of salmonellosis in CZ in 2012–2023 and identify areas for improving surveillance and public health actions.

**METHODS:**

We extracted data on notified salmonellosis cases from the national surveillance system and analysed the dataset for demographic characteristics, hospitalisation, transmission mode and suspected vehicle, descriptively and by chi-square tests and logistic regression.

**RESULTS:**

In 2012–2023, 130,990 cases (102.7/100,000 inhabitants) were notified. The overall annual notification rate decreased from 111.7 per 100,000 in 2012–2017 to 94.2 per 100,000 in 2018–2023. Most cases were children and adolescents aged < 15 years (n = 68,370; 52.2%) and most were females (n = 68,425; 52.2%) but males dominated among cases aged < 15 years (35,790/68,370; 52.3%). Few cases were imported (n = 2,627; 2.0%) or outbreak-related (n = 5,361; 4.1%); most were diagnosed in June–October (n = 83,057; 63.4%). Of all notified cases, 22.2% (n = 29,082) were hospitalised. Age of ≥ 40 years was associated with hospitalisation and *Salmonella* sepsis (p < 0.001). Transmission mode was identified for 59,729 (45.6%) cases.

**CONCLUSION:**

Despite a decline, the notification rates of salmonellosis remain high in CZ. The effectiveness of the surveillance system is reflected in relatively low hospitalisation rates compared with some other European countries, yet the source and transmission mode were unidentified in over half of the cases. We encourage enhancing epidemiological investigations, improving sample submission to the National Reference Laboratory and molecular surveillance.

**Key public health messages**

**What did you want to address in this study and why?**

Salmonellosis is a common food-borne disease, affecting mainly children and adolescents. Despite a decline, Czechia has five times more salmonellosis cases than the average of the other European countries. We analysed national data to identify trends, distribution changes, hospitalisation risk and transmission modes to improve public health preventive actions.

**What have we learnt from this study?**

We discovered that 52% of salmonellosis cases in Czechia were children and adolescents under 15 years, but the risk of complications like sepsis and hospitalisation is higher in adults aged older than 40 years. Most infections were acquired in Czechia and were not part of identified outbreaks. One in five cases was hospitalised. More than half of the cases did not identify transmission modes and suspected food.

**What are the implications of your findings for public health?**

The completeness of some surveillance data is limited. Enhancing the identification of sources and transmission modes during epidemiological investigations is crucial. We recommend improving sample submission to the National Reference Laboratory, continuous public education, and promoting responsibility across industry and authorities.

## Introduction

Non-typhoidal salmonellae (e.g. *Salmonella* Enteritidis, *S.* Typhimurium, *S.* Infantis) are major food-borne pathogens causing salmonellosis worldwide. Salmonellosis, after an incubation period of 12–36 h, often manifests with mild, self-limiting gastrointestinal symptoms, like diarrhoea, abdominal pain and fever. Severe systemic infections (bacteraemia, meningitis and osteomyelitis) may occur, especially in infants aged < 3 months, older adults, pregnant people or people with weakened immune systems [1]. Long-term complications, such as reactive arthritis or irritable bowel syndrome, may develop in some patients. Diagnosis is based on the medical history and analysis of clinical specimens. Treatment focuses on rehydration, electrolyte replacement and nutritional management. Hospitalisation is rarely needed. *Salmonella* shedding lasts ca 4 weeks in adults and up to 7 weeks in children, occasionally longer [2].

Despite harmonised European Union (EU) control programmes for poultry, *Salmonella* remains a leading cause of food-borne outbreaks in Europe [3]. About 86% of human *Salmonella* infections are estimated to originate from food, while mass production and global distribution of food enable rapid transmission of pathogens [4]. Most common vehicles for *Salmonella* infections are eggs and poultry meat, followed by pork meat and fresh green products such as salad or other vegetables [3,5]. Approximately 30% of human salmonellosis cases in the EU may be attributed to the pig reservoir [6]. Cross-contamination may occur at all stages of food production as *Salmonella* can tolerate various environmental conditions [7]. However, other transmission routes exist. An estimated 9–10% of notified *Salmonella* cases have been attributed to direct or indirect contact with animals, including pets, livestock and their environments [8]. Climate change and global warming contribute to an increase in salmonellosis cases [9]. In Czechia (CZ), for every 1°C increase in temperature, a significant 6.2% rise in salmonellosis cases was observed [10].

The strategies for reduction and control of *Salmonella* in poultry were implemented in CZ in 2008 [11]. Good hygiene and high biosecurity are the main measures to prevent introduction and spread of pathogens in feed mills and on farms [11]. Vaccination of poultry and pigs is an additional option to control *Salmonella,* mainly targeting *S.* Enteritidis and Typhimurium [12].

A significant decrease in human cases of salmonellosis was recorded in CZ between 2008 and 2014. Since then and until 2022, the notification rate has remained stable. Nonetheless, the rate was five times greater than the EU average: 71.9 vs 15.3 per 100,000 population in 2022, respectively [5]. We aimed to describe trends, basic epidemiological characteristics, transmission modes and vehicles of *Salmonella* infections in human population of CZ. Further, we compared two periods (2012–2017 and 2018–2023) to determine the changes.

## Methods

### Surveillance of salmonellosis

**Figure 1:**
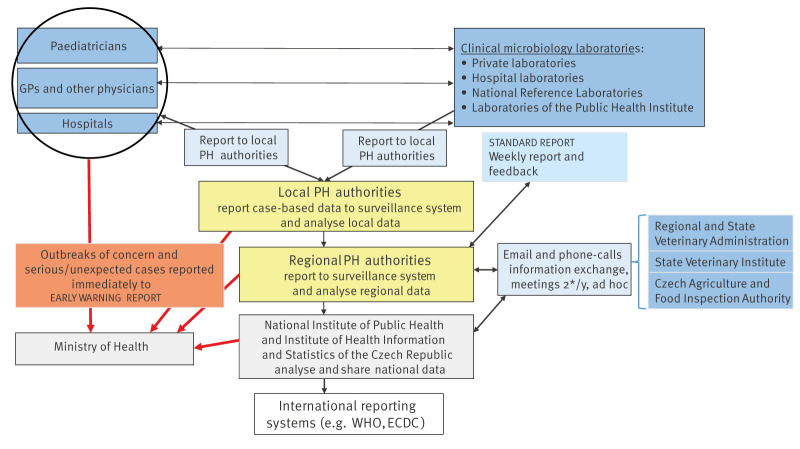
Surveillance system of communicable diseases, including salmonellosis, Czechia

GP: general practitioner; PH: public health; ECDC: European Centre for Disease Prevention and Control; WHO: World Health Organization.

In CZ, salmonellosis is mandatorily notifiable under the Act Number 258/2000 Coll. on Public Health Protection [13]. The nationwide surveillance system complies with the EU requirements of the Commission Implementing Decision (EU) 2018/945 [14]. Since January 2018, the reporting system has been electronical (ISIN). Physicians and clinical microbiology laboratories diagnosing a case notify the regional public health authorities (RPHA) who collect case data, conduct epidemiological investigations and upload relevant information into ISIN (Figure 1). All laboratory results (isolates) from a patient's course of illness are consolidated under a single case.

In 2023, according to the Czech Statistical Office, there were 160 acute care hospitals and 25,478 independent physician surgeries in CZ, including 5,473 general practitioners for adults and 2,120 general practitioners for children and adolescents. The Czech Institute for Accreditation registered 240 clinical microbiology laboratories (as of 12 February 2025) of which 118 participate in the external quality assessment (EQA) organised by the National Reference Laboratory for *Salmonella* (NRL). The EQA includes an educational sample containing *Salmonella*. The participating laboratories either identify the serovars by themselves or forward the isolates to the NRL.

Upon receiving case information, RPHA interview the patient or their family in cases of minors and persons unable to respond, using brief regional epidemiological investigation questionnaires covering epidemiologically relevant details, including questions on possible modes of transmission and potential exposures. The proportion of interviewed cases is not known. Additionally, uniform hypothesis-generating questionnaires for atypical *Salmonella* serovars (other than *S.* Enteritidis and Typhimurium) focus on potential vehicles in more detail. However, these questionnaires have not yet been integrated electronically into the ISIN and were therefore not included in our study.

Cases are classified as probable and confirmed according to the EU case definition [14] and outbreaks according to the World Health Organization (WHO) definition [15]. Outbreak-related isolates are considered genetically linked if they fall within ≤ 5 allele differences based on core genome multilocus sequence typing (cgMLST). All cases are reported as case-based data, regardless of whether they are sporadic or outbreak-related. Once uploaded, data are stored under code A02 (Other *Salmonella* infections) according to the International Statistical Classification of Diseases, 10th Revision (ICD-10) [16]. The National Institute of Public Health collaborates on data validation, analysis and international reporting.

### Laboratory data

Clinical microbiology laboratories can identify ca 5–10 of the most common *Salmonella* serovars (assumption based on summaries from the national EQAs). The NRL performs serotyping of less common serovars and confirmatory testing upon request. Molecular methods, such as pulsed-field gel electrophoresis, multiple-locus variable number tandem repeat analysis, cgMLST or other whole genome sequencing (WGS) methods, are also used. To enhance detection of clusters and outbreaks, since 2023, laboratories must submit representative *Salmonella* isolates to the NRL: every tenth *S.* Enteritidis, every fifth *S.* Typhimurium and every third *S.* Infantis. During outbreaks, at least four isolates (if available) must be submitted, along with all isolates from other specimen types than from the gastrointestinal tract or the urinary system.

### Descriptive analysis

We extracted data on all probable and confirmed salmonellosis cases diagnosed in 2012–2023 and pseudonymised the dataset. We included information on basic epidemiological characteristics, e.g. sex, age, geographical distribution of cases by residence, seasonality (based on notification date), outbreak, complication, hospitalisation, death, importation status, transmission mode and vehicle or source. Information on the vehicle or source was derived from patient interviews, outbreak investigations, laboratory testings or a combination of these methods. Testing of food samples was performed during outbreaks if samples were available.

A comparative analysis of selected variables (age, imported vs autochthonous, hospitalisation, outbreak and vehicle) was conducted for 2012–2017 and 2018–2023. Clinical conditions defined by an ICD-10 code (e.g. A02.1 for *Salmonella* sepsis) were only available from 2018 onwards, therefore the calculations were done only for the second period. In the first period, only the total number of outbreak-related cases per year was recorded, whereas in the second period, data were available for each outbreak.

Notification rates were calculated using Czech Statistical Office mid-year population data (1 July). Data were processed using Excel (2016) and Stata, version 17 (Stata, College Station, the United States (US).

### Statistical analysis

Associations between the study variables were tested using Pearson’s chi-square test, linear and logistic regression. The likelihood of hospitalisation and of developing sepsis was assessed. Based on the univariate analysis, the significant predictors (age group, sex, region and quarter year; period only for hospitalisation) were entered into multiple logistic regression models.

The results are expressed as odds ratios (OR) and 95% confidence intervals (CI). Statistical significance was set at p < 0.05.

## Results

A total of 130,990 salmonellosis cases were notified: 70,512 in 2012–2017 and 60,478 in 2018–2023. The overall annual notification rate per 100,000 inhabitants was 102.7, decreasing from 111.7 in 2012–2017 to 94.2 in 2018–2023. Case classification was available only for the second period, with 59,087 (97.7%) classified as confirmed and 1,391 (2.3%) as probable.

**Figure 2:**
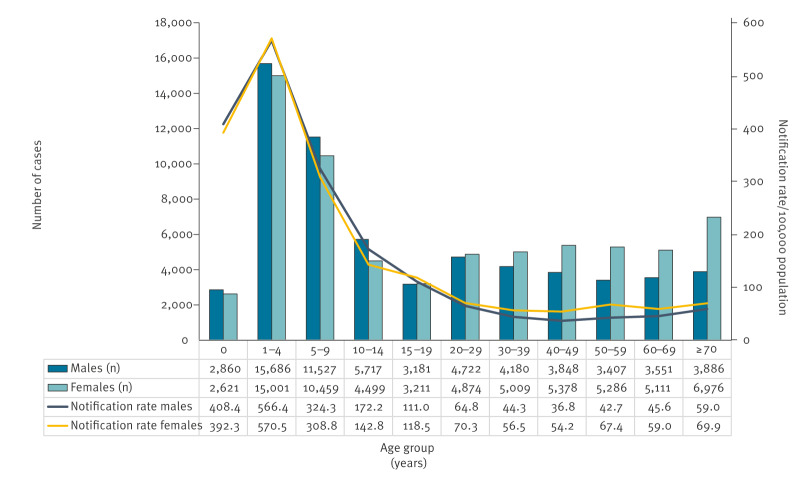
Notification rate of salmonellosis, by age and sex, Czechia, 2012–2023 (n = 130,990)

The ages of the cases ranged from 0 to 102 years (mean: 25.1 years; median: 13 years (lower and upper quartiles (Q1, Q3): 4, 44]). Most cases were children and adolescents aged < 15 years (n = 68,370; 52.2%) and of these, 5,481 (8.0%) were infants aged < 1 year (Figure 2). In 2022, children and adolescents aged < 15 years still constituted 52.5% (4,035/7,681) of the cases and in 2023, 49.2% (3,795/7,706). The main characteristics of salmonellosis cases reported within national surveillance system are presented in Supplementary Table S1.

**Figure 3:**
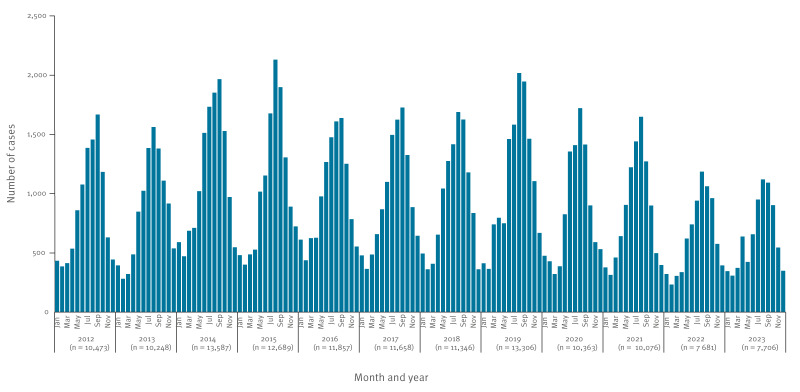
Notified cases of salmonellosis, by month of symptom onset, Czechia, 2012–2023 (n = 130,990)

Most cases were females (n = 68,425; 52.2%) in all years except in 2022. When stratified by age, males dominated among cases aged < 15 years (35,790/68,370; 52.3%) (Figure 2). Most (n = 83,057; 63.4%) cases were diagnosed between June and October. The median calendar week of reporting was 31 (Q1, Q3: 22, 39). A downward yearly trend in case numbers was observed (p < 0.001) (Figure 3).

Geographically, the highest 12-year notification rates were recorded in the Vysočina (145.4/100,000), Pardubice (145.4/100,000) and South Bohemia (144.8/100,000) regions.

### Outcome and clinical presentation

**Figure 4:**
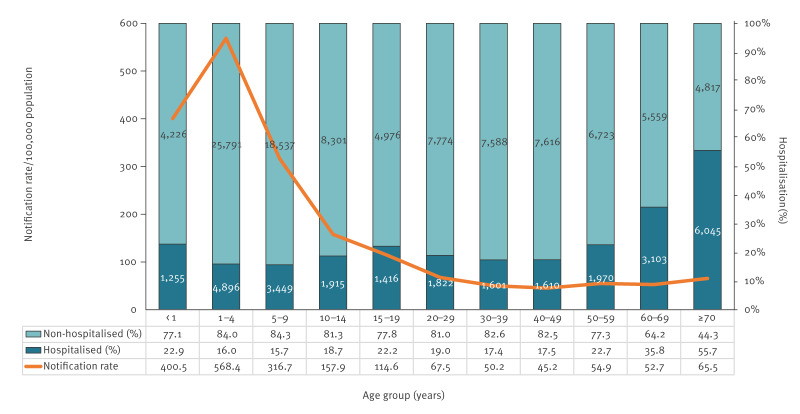
Notification rate of salmonellosis and proportion of hospitalised cases, by age group, Czechia, 2012–2023 (n = 130,990)

Of all notified cases, 22.2% (n = 29,082) required hospitalisation. Hospitalisation proportions were similar across the two periods: 22.9% and 21.4%, respectively. Among those hospitalised, 6,045 (20.8%) were aged ≥ 70 years, 4,896 (16.8%) were 1–4 years, 3,449 (11.8%) were 5–9 years and 3,103 (10.7%) were 60–69 years. The highest hospitalisation rate (6,045/10,682; 55.7%) was seen in cases aged ≥ 70 years (Figure 4).

A total of 173 cases (0.1%) died, 33 deaths were directly attributable to salmonellosis.

From 2018 to 2023, of the 60,478 cases with information on clinical conditions, 59,544 (98.5%) were coded as A02.0 (*Salmonella* enteritis), 386 (0.6%) as A02.1 (*S*. sepsis), 125 (0.2%) as A02.2 (localised *S*. infections), 339 (0.6%) as A02.8 (other specified *S*. infections) and 84 (0.1%) as A02.9 (*S*. infection, unspecified). Sepsis was most common in the oldest age groups: ≥ 70 years (193/5,281; 3.7%) and 60–69 years (80/3,874; 2.1%), altogether accounting for 70.7% of all 386 sepsis cases. Of the sepsis cases, 52 (13.5%) died, including one infant and 51 adults (range: 45–92 years; mean age 71.8; median 74 years). Sepsis was the direct cause of death in 23 cases. In the other 29, death was caused by various conditions (e.g. other than *Salmonella* sepsis, cardiac arrest, pneumonia or organ failure), though these were likely influenced by the underlying *Salmonella* sepsis.

### Importation and transmission mode

Of all the cases, 2,627 (2%) were considered imported. The number of imported cases per year fluctuated during the study period, with the highest counts observed in 2019 followed by a steep decline in 2020 and 2021. When comparing the two periods (2012–2017 and 2018–2023), the proportions of imported cases were 1.8% and 2.2%, respectively. Countries with more than 100 imported cases in the first period were Croatia (n = 140), Slovakia (n = 126), Türkiye (n = 118) and Egypt (n = 111), and in the second period, Türkiye (n = 212), Egypt (n = 210) and Tunisia (n = 116).

The reported transmission mode, based on epidemiological investigations and mostly as identified by the cases, was specified in 59,729 cases (45.6% of total cases; 42.1% and 49.6% in the two periods). In 96.9% of these cases, the transmission was considered food-borne (n = 57,852), followed by human-to-human contact (n = 1,745; 2.9%) and waterborne (n = 111; 0.2%). Among food items, specified in 94.6% of food-borne transmissions (n = 54,755), the most common vehicles were eggs (38.5%), poultry meat (21.0%) and ready-to-eat food (11.8%). Other reported vehicles included confectionery (6.4%), sausages (5.5%), other meat products (5.3%), deli products (3.6%), ice cream (1.9%), fish products (1.5%), mayonnaise (1.2%), fruit (1.2%), dairy (1.0%), vegetables (0.6%) and others (0.5%).

A total of 5,361 cases (4.1%; range: 2.2% in 2020 and 6.6% in 2023) were linked to outbreaks. In the first period, 3,069 (4.4%) outbreak-related cases were reported, while in the second period, 2,292 cases (3.8%) were recorded in 94 outbreaks, mostly of *S.* Enteritidis. In these outbreaks, 3,544 (66.1%) cases were attributed to food, 70 (1.3%) to human-to-human contact, 27 (0.5%) to water and for 1,716 (32.0%) cases the vehicle could not be identified. Among food-borne outbreak cases, the most common vehicles were ready-to-eat foods (1,517; 42.8%), confectionery (556; 15.7%), eggs (347; 9.8%) and poultry meat (259; 7.3%).

### Laboratory data

*Salmonella* was detected by culture method in 58,684 (44.8%) cases, and the detection method was unspecified in 55.2% cases. Throughout the study period, *S.* Enteritidis was the most common serovar (n = 112,466; 85.9%), followed by *S.* Typhimurium (monophasic variants excluded) (n = 7,158; 5.5%) (Table 1). *Salmonella* Infantis was the third or fourth most common serovar (n = 1,146; 0.9%) each year. In 2018, *S.* Coeln emerged, and in 2023, *S.* Thompson became prominent, with 42 and 99 outbreak-related cases, respectively. Since 2015, monophasic *S.* Typhimurium has been among the six most common serovars, with 29 outbreak cases in 2021.

**Table 1:** Distribution of the six most common *Salmonella* serovars, Czechia, 2012–2023 (n = 130,990)

Serovar	n	%	Serovar	n	%	Serovar	n	%	Serovar	n	%	Serovar	n	%	Serovar	n	%
A. 2012–2017 (n = 70,512)																	
2012			2013			2014			2015			2016			2017		
Enteritidis	9,008	86	Enteritidis	8,721	85.1	Enteritidis	12,117	89.2	Enteritidis	10,960	86.4	Enteritidis	10,471	88.3	Enteritidis	9,642	82.7
Typhimurium	711	6.8	Typhimurium	670	6.5	Typhimurium	671	4.9	Typhimurium	935	7.4	Typhimurium	593	5	Typhimurium	636	5.5
Infantis	118	1.1	Infantis	113	1.1	Infantis	98	0.7	Infantis	94	0.7	Infantis	110	0.9	Bareilly	106	0.9
Agona	51	0.5	Agona	63	0.6	Indiana	64	0.5	Serogroup 0:9 (D)	51	0.4	Virchow	96	0.8	Infantis	94	0.8
Stanley	38	0.4	Indiana	44	0.4	Ohio	46	0.3	Virchow	46	0.4	Monophasic T.	41	0.3	Monophasic T.	47	0.4
Virchow	32	0.3	Stanley	44	0.4	Serogroup 0:9 (D)	38	0.3	Monophasic T.	35	0.3	Serogroup 0:4 (B)	23	0.2	Serogroup 0:9 (D)	23	0.2
Other	515	3.9	Other	593	4.6	Other	553	3.2	Other	568	3.6	Other	523	3.7	Other	1,110	8.9
Total	10,473	100	Total	10,248	100	Total	13,587	100	Total	12,689	100	Total	11,857	100	Total	11,658	100
B. 2018–2023 (n = 60,478)																	
2018			2019			2020			2021			2022			2023		
Enteritidis	9,553	84.2	Enteritidis	11,481	86.3	Enteritidis	9,085	87.7	Enteritidis	8,897	88.3	Enteritidis	6,349	82.7	Enteritidis	6,182	80.2
Typhimurium	657	5.8	Typhimurium	527	4	Typhimurium	374	3.6	Typhimurium	495	4.9	Typhimurium	468	6.1	Typhimurium	421	5.5
Monophasic T.	93	0.8	Infantis	115	0.9	Infantis	101	1	Monophasic T.	109	1.1	Monophasic T.	81	1.1	Thompson	145	1.9
Infantis	88	0.8	Coeln	86	0.6	Monophasic T.	77	0.7	Infantis	74	0.7	Infantis	65	0.8	Infantis	76	1
Coeln	87	0.8	Bareilly	78	0.6	Coeln	45	0.4	Coeln	64	0.6	Coeln	56	0.7	Monophasic T.	74	1
Bareilly	66	0.6	Monophasic T.	73	0.5	Kentucky	40	0.4	Newport	20	0.2	Newport	32	0.4	Coeln	71	0.9
Other	802	6.1	Other	946	6	Other	641	5.5	Other	417	3.5	Other	630	7	Other	737	8.3
Total	11,346	100	Total	13,306	100	Total	10,363	100	Total	10,076	100	Total	7,681	100	Total	7,706	100

Monophasic T.: monophasic *Salmonella* Typhimurium.

### Comparing 2012–2017 and 2018–2023 data

Comparison brought up the following results: the mean age of salmonellosis cases was significantly lower in the first period (24.8 vs 25.5 years; p < 0.001). Case numbers significantly declined between 2012–2017 and 2018–2023 in all regions except for three (p < 0.001). A decline was also observed when stratified by quarter years (p < 0.001). No differences were seen between sexes (p = 0.094). Age, sex, study period (2012–2017 vs 2018–2023), region and quarter year (October–December) were associated with hospitalisation (Table 2). Cases aged < 1 year and ≥ 40 years had higher odds of hospitalisation and developing sepsis. The likelihood of developing *S.* sepsis was also significantly associated with male sex, region and quarter year (July–September).

**Table 2:** Multiple logistic regression analysis of hospitalisation and sepsis of cases with *Salmonella* infection, Czechia, 2012–2023 (n = 130,990)

Characteristic	Hospitalisation (2012–2023; n = 130,990)			Sepsis (2018–2023; n = 60,478)		
	aOR^a^	95% CI	p value	aOR^a^	95% CI	p value
Age group (years)	< 0.001			< 0.001		
0	1.61	1.49–1.73	< 0.001	9.96	3.17–31.32	< 0.001
1–4	1.02	0.98–1.07	0.358	2.52	0.83–7.65	0.104
5–9^b^	Reference			Reference		
10–14	1.25	1.18–1.33	< 0.001	3.13	0.91–10.69	0.069
15–19	1.54	1.44–1.66	< 0.001	3.07	0.77–12.30	0.113
20–29	1.26	1.18–1.34	< 0.001	6.86	2.18–21.56	0.001
30–39	1.14	1.07–1.22	< 0.001	2.79	0.70–11.15	0.148
40–49	1.14	1.07–1.22	< 0.001	13.46	4.63–39.11	< 0.001
50–59	1.58	1.49–1.68	< 0.001	25.79	9.16–72.55	< 0.001
60–69	3.02	2.85–3.20	< 0.001	57.50	21.03–157.19	< 0.001
≥ 70	6.87	6.51–7.24	< 0.001	107.51	39.88–289.84	< 0.001
Sex						
Female	Reference			Reference		
Male	1.07	1.04–1.10	< 0.001	2.52	2.05–3.12	< 0.001
Period (years)						
2012–2017	Reference			Reference		
2018–2023	0.88	0.86–0.90	< 0.001	NA	NA	NA
Region	< 0.001			< 0.001		
Region of the City of Prague	Reference			Reference		
Central Bohemian region	1.05	0.98–1.12	0.197	0.57	0.38–0.87	0.010
South Bohemian region	1.16	1.08–1.25	< 0.001	0.69	0.45–1.05	0.085
Plzeň region	1.60	1.49–1.72	< 0.001	0.86	0.55–1.35	0.503
Karlovy Vary region	1.22	1.10–1.36	< 0.001	1.14	0.64–2.03	0.667
Ústí nad Labem region	1.50	1.38–1.62	< 0.001	0.49	0.27–0.89	0.018
Liberec region	1.53	1.39–1.68	< 0.001	1.08	0.63–1.84	0.780
Hradec Králové region	1.09	1.01–1.18	0.026	0.40	0.23–0.71	0.002
Pardubice region	1.17	1.08–1.26	< 0.001	0.31	0.18–0.56	< 0.001
Vysočina region	1.42	1.32–1.52	< 0.001	0.34	0.20–0.61	< 0.001
South Moravian region	1.09	1.02–1.16	0.013	0.33	0.20–0.54	< 0.001
Olomouc region	1.05	0.97–1.14	0.205	0.37	0.21–0.64	< 0.001
Zlín region	1.47	1.36–1.59	< 0.001	0.43	0.24–0.75	0.003
Moravian-Silesian region	1.22	1.14–1.31	< 0.001	0.40	0.26–0.63	< 0.001
Quarter year	< 0.001			< 0.001		
Jan–Mar	Reference			Reference		
Apr–Jun	0.96	0.92–1.01	0.110	0.71	0.51–0.97	0.032
Jul–Sep	0.97	0.93–1.01	0.157	0.45	0.33–0.61	< 0.001
Oct–Dec	0.91	0.87–0.96	< 0.001	0.71	0.52–0.97	0.031

aOR: adjusted odds ratio; CI: confidence interval; NA: not available.

^a^ The adjustment was done for influence of all other independent variables included in the model.

^b^ This age group was chosen as a reference group based on them being substantially represented and having the lowest proportion of hospitalisation and sepsis.

## Discussion

Salmonellosis is the second most notified zoonosis in the EU [5]. In 2022, the average notification rate was 15.3 cases per 100,000 population, highest in CZ (71.9). This disparity has been observed long-term, for example in 2013 and 2017, when the EU average was 20.3 and 19.7, and the notification rate in CZ was 93.1 and 108.5, respectively [17]. Notification rates between EU countries differ and the reasons are not well elucidated but may stem from differing case definitions and variations in reporting systems, incomplete population coverage and other factors, like disease prevalence in the food‐producing animal population and in food and animal trade between countries, as suggested in the European Union One Health 2022 Zoonoses Report [5]. Under-ascertainment and under-reporting may be additional reasons [18,19]. High notification rates in CZ partly reflect the country’s infectious disease surveillance system, which has been operating continuously since 1982 and that clinicians and laboratories are complying to mandatory reporting. Therefore, patients with suspected *Salmonella* infections are tested and notified rather than overlooked. In CZ, *Salmonella* was the leading cause of acute gastroenteritis until 2006, then overtaken by *Campylobacter* in 2007 [10]. The transition to electronic reporting (2018) did not markedly change the notified incidence. The trend of salmonellosis has been declining since 2020. COVID-19 pandemic restrictions reduced also notified cases of other food- and waterborne infections, including travel-related and domestically acquired cases [20].

Children under 5 years are more susceptible to *Salmonella* infections due to their immature immune systems and more permeable gastrointestinal tracts [21]. In our study, the highest notification rates were among children aged 1–4 years (568.4/100,000) and < 1 years (400.5/100,000), which is 6–7 times higher than the EU average for 0–4-year-olds in 2022 and 2020 (81.5 and 76.3, respectively) [22,23]. We also observed high rates in children and adolescents aged 5–19 years. This could be due to a higher proportion of symptomatic infections and an increased likelihood of parents seeking medical care for their children [22]. The sex differences in the notification rate for salmonellosis [24,25] are not fully understood, but genetic, hormonal and gut microbiota interactions could be possible contributors [24].

More *Salmonella* cases are notified during the summer months in the EU countries [5]. Elevated temperatures promote *Salmonella* proliferation in inadequately refrigerated foods [26]. Outdoor food preparation may involve suboptimal hygiene and temperature control, increasing cross-contamination risk. Despite peak summer travels, travel-related cases in CZ remain low [20]. Heat stress in poultry can increase intestinal permeability and *Salmonella* translocation, raising contamination risk during slaughter [27,28]. In CZ, the peak in imports of chicken meat and eggs (2012–2023) occur during pre-Easter and pre-Christmas time [29], thus, we assume imports did not have a direct effect on the summer peak of human cases.

The average hospitalisation rate for salmonellosis in our study was 22.2%, highest in individuals aged ≥ 70 years (20.8%), 1–4 (16.8%), 5–9 (11.8%) and 60–69 years (10.7%). In contrast, average rates in the EU countries reporting hospitalisation are higher, with 38.9% of cases hospitalised in 2022 [5]. Rates vary widely across countries with the highest proportions of hospitalised cases in countries with low notification rates (5.3–7.3%), suggesting these countries primarily capture severe cases. Lower hospitalisation rate in CZ suggests a robust surveillance system that detects even milder infections. The odds of hospitalisation and *Salmonella* sepsis increased significantly with age (≥ 40 years; p < 0.001), consistent with established risk factors such as age-related immune changes, comorbidities and overall reduced physiological resilience [21].

Data from 2016 to 2021 show that imported cases in CZ represented only < 3% of all cases [20]. According to our study, 98% of cases were locally acquired, compared with 62% in the EU in 2022 [5]. The continuing decline may reflect increased public awareness and improved food handling practices following reported outbreaks such as *S.* Bareilly [30], *S.* Strathcona [31] and *S.* Mbandaka [32]. In the EU, *Salmonella* accounted for 17.6% of reported food-borne outbreaks in 2022, with common vehicles including eggs, mixed foods, pork, sweets, chocolate and bakery products [5]. In our study, only 4.1% of the cases were linked to outbreaks, which may seem low, but CZ notification rate of outbreak-related cases was average in the EU [5]. In 2022, the EU marked the first year when most reported food-borne outbreaks occurred in restaurants, pubs and takeaway services [5]. The US Centers for Disease Control and Prevention (CDC) estimated that 59% of food-borne outbreaks in the US occurred in the food service sector with 75% of these cases attributed to improper food handling in restaurants [33]. The Czech reporting system does not collect information on food purchase points, but this information is asked during outbreak investigations. Compared with some other EU countries, there is a lack of microbiologically confirmed evidence for sources or vehicles of food-borne outbreaks in CZ [5].

We identified transmission pathway in only 45% of cases. The most common vehicles according to the study were eggs, poultry meat and ready-to-eat foods. Both domestic (mainly eggs) and imported (mainly poultry meat and pork) food products contribute to *Salmonella* cases in CZ [29,34]. Imported products, especially from countries with different food safety standards, may pose risks despite strict controls. Monitoring of zoonotic *Salmonella* spp. in the EU includes cattle, pigs, broilers, turkeys, and other animals under Regulation (EU) 2019/627, with additional testing by Czech Agriculture and Food Inspection Authority as part of the national food control programme.

The analysis of human *Salmonella* serovars from 2012 to 2023 showed consistent dominance of *S.* Enteritidis, followed by *S.* Typhimurium and *S.* Infantis. Typhimurium, in particular, has a broad reservoir range, including many species raised for food and/or kept as household pets [6]. Monophasic *S.* Typhimurium notably increased in CZ from 2016 onwards. These trends align with the EU data, where in 2022, *S.* Enteritidis (67.3%), *S.* Typhimurium (13.1%), monophasic *S.* Typhimurium (1,4,[5],12:i:−) (4.3%) and *S.* Infantis (2.3%) were predominant [5]. Local and cross-border outbreaks, such as of *S.* Bareilly in 2017–2018 and *S*. Thompson in 2023, along with changes in food consumption patterns and international trade, influence serovar dynamics [6].

Sequencing methods are increasingly used in outbreak investigations in many countries, especially in Western Europe [30,35]. However, in CZ, they are only employed for certain internationally recognised outbreaks. Although efforts have been made to routinely sequence a representative sample of isolates at the NRL, estimating compliance remains challenging and only 161 of the 240 registered clinical microbiology laboratories reported results. We observed an increase in the proportions of cases confirmed by NRL from 2020 to 2023 (1.0–4.1%).

Under-reporting exists in any surveillance system, but ca 10,000 cases were annually reported in CZ. Compared with the EU data, this suggests that our system comprises even milder cases. Variability in surveillance quality across regions and periods could also impact data comparability. No considerable changes in case identification occurred during the study, except for the inclusion of WGS for some isolates. While we did not evaluate completeness, Liptáková et al. [36] estimated 98.8% sensitivity for technically identical measles surveillance system, suggesting similar reliability for salmonellosis surveillance. Nonetheless, as 98% of cases in the study were reported as confirmed, but only 45% detected by culture method, we assume that the remaining cases with unspecified laboratory methods were also identified through culture and consider this to be a reporting limitation. Inconsistent reporting may hinder detection of outbreaks. Although outbreaks can be identified and/or confirmed based on WGS data, the scale of sequencing in CZ remains limited. Food-borne illnesses are often difficult to trace, particularly with multiple potential transmission sources. Recall bias is expected when interviewing patients regarding food consumption. Information on transmission mode or food vehicle was obtained through case interviews, with confirmation through outbreak investigation or laboratory testing possible in only a minority of cases. Further limitation was our inability to estimate the proportion of salmonellosis cases attributable to direct or indirect animal contact, as this transmission route was not specifically captured in ISIN.

## Conclusion

Salmonellosis in CZ is decreasing but remains well above the EU average, highlighting the ongoing public health burden. The effectiveness of the surveillance system is reflected in relatively low hospitalisation rates, yet the source and transmission remain unknown for over half of the cases. Strengthening outbreak investigations, including analytical epidemiological studies when microbiological proof is lacking and more frequent WGS of isolates, and improving data quality are essential for guiding targeted control measures. Ensuring food safety requires collaboration across public health, veterinary, and food sectors, along with continuous public education and awareness. These efforts are critical to reduce transmission and protect population health.

## Data Availability

All data presented in the manuscript or supplementary material are available from the corresponding author upon reasonable request.

## Supplementary Data

152000 Supplementary Material
