# Supplementary material for: Epidemiology of human salmonellosis in Czechia ─ a country with the highest European notification rate, 2012 to 2023
Source: Euro Surveill. 2026 Jan 22;31(3):2500223. doi: 10.2807/1560-7917.ES.2026.31.3.2500223 (PMC12848985; doi:10.2807/1560-7917.ES.2026.31.3.2500223)
Supplement: Supplementary Material [file 25-00223_SPACKOVA_Supplement.pdf]

Supplementary table S1. Main characteristics of salmonellosis cases reported within the Czech Republic surveillance system from 2012 to 2017 and 2018 to 2023[#]

		1st period (2012-2017)							2nd period (2018-2023)						
		2012	2013	2014	2015	2016	2017	Total"	2018	2019	2020	2021	2022	2023	Total"
**Salmonellosis cases (n=130,990)**		10473	10248	13587	12689	11857	11658	70512	11346	13306	10363	10076	7681	7706	60478
	% of total for respective period	14,9	14,5	19,3	18,0	16,8	16,5	100	18,8	22,0	17,1	16,7	12,7	12,7	100
	% of confirmed cases[&]	n.a.	n.a.	n.a.	n.a.	n.a.	n.a.	n.a.	96,1	97,7	98,4	99,1	98,3	96,5	97,7
	% of outbreak cases[^]	3,8	2,4	6,0	4,8	4,8	3,8	4,4	2,4	5,8	2,2	2,3	3,7	6,6	3,8
**- by quarter**	I	1178	993	1677	1352	1605	1283	8088	1158	1342	1341	1110	904	1008	6863
	II	2332	2153	3071	2541	2764	2532	15393	2656	2705	2239	2601	1620	1642	13463
	III	4378	4334	5514	5506	4702	4690	29124	4451	5455	4320	4263	3037	2961	24487
	IV	2585	2768	3325	3290	2786	3153	17907	3081	3804	2463	2102	2120	2095	15665
**Males (n=62,565)**		4988	4847	6360	6186	5580	5567	33528	5422	6361	4898	4844	3792	3720	29037
	age, mean ± SD	21.2 ± 23.6	20.9 ± 23.4	20.4 ± 23.1	22.2 ± 23.6	22.2 ± 23.9	23.0 ± 23.9	21.7 ± 23.6	21.9 ± 23.6	22.4 ± 23.8	22.5 ± 24.4	22.3 ± 24.2	22.9 ± 25.0	23.5 ± 24.4	22.5 ± 24.2
	age, median (Q1 - Q3)[$]	9 (3-36)	9 (3-35)	9 (3-34)	11 (4-37)	10 (4-37)	11 (4-39)	10 (4-36)	10 (4-37)	11 (4-38)	11 (3-39)	11 (4-38)	11 (4-40)	13 (4-40)	11 (4-39)
	% of total	47,6	47,3	46,8	48,8	47,0	47,7	47,5	47,8	47,8	47,3	48,1	49,4	47,8	48,0
**Females (n=68,425)**		5485	5401	7227	6503	6277	6091	36984	5924	6945	5465	5232	3889	3986	31441
	age, mean ± SD	26.9 ± 26.3	27.7 ± 26.4	27.0 ± 26.4	27.5 ± 26.1	28.8 ± 26.9	28.0 ± 26.1	27.6 ± 26.4	28.2 ± 26.7	27.9 ± 26.6	28.3 ± 27.3	27.1 ± 26.8	28.5 ± 27.6	29.7 ± 27.5	28.2 ± 27.0
	age, median (Q1 - Q3)[$]	17 (4-48)	19 (4-49)	16 (4-48)	17 (4-49)	19 (5-51)	18 (5-49)	17 (4-49)	17 (5-51)	16 (4-50)	16 (4-52)	15 (4-49)	16 (4-52)	19 (5-53)	16 (4-51)
	% of total	52,4	52,7	53,2	51,3	52,9	52,3	52,5	52,2	52,2	52,7	51,9	50,6	51,7	52,0
**Specific notification rates**															
**- overall**		99,7	97,5	129,1	120,4	112,2	110,1	111,7	106,8	124,7	96,8	96,0	71,4	71,2	94,2
**- by sex**	males	96,6	93,9	123,0	119,4	107,5	106,9	108,1	103,7	121,0	92,9	93,6	71,8	70,1	91,8
	females	102,6	101,0	134,9	121,3	116,8	113,2	115,1	109,8	128,3	100,7	98,2	71,0	72,2	96,6
**- by age groups**	0	436,0	416,7	501,0	467,6	394,1	376,6	438,1	414,8	491,3	431,3	459,1	323,1	286,4	395,0
	1-4	586,5	577,0	744,6	649,7	590,5	575,4	621,6	561,9	659,3	521,9	534,8	390,1	369,7	502,6
	5-9	329,3	312,3	455,7	381,6	360,9	329,2	361,9	338,6	379,9	284,6	283,6	223,5	204,6	290,4
	10-14	130,7	121,5	199,4	187,3	186,4	199,1	175,8	184,7	211,7	167,7	153,6	102,8	105,2	151,0
	15-19	84,3	80,8	123,0	132,9	118,2	116,7	110,1	120,7	149,0	112,5	117,0	84,7	75,5	112,8
	20-29	57,1	60,7	75,5	81,1	70,8	71,9	68,0	68,2	80,9	61,9	65,1	42,7	53,2	61,9
	30-39	50,6	47,5	56,9	58,6	56,4	59,1	53,9	48,4	57,4	39,8	42,0	29,3	37,1	42,1
	40-49	44,4	49,2	59,1	56,3	51,3	52,0	53,1	47,7	60,1	42,5	41,2	30,2	32,0	41,6
	50-59	51,2	50,7	66,6	64,5	58,2	58,7	58,2	55,6	66,6	50,9	46,0	36,4	37,0	49,1
	60-69	49,5	47,2	61,3	62,5	58,0	60,1	56,4	59,0	61,0	52,9	49,9	39,5	39,5	50,1
	70+	70,4	66,7	82,8	75,8	79,7	68,6	75,5	67,4	80,4	65,5	59,5	50,0	49,0	62,1
**- by regions**	Region of the City of Prague	60,6	53,2	73,3	74,3	78,3	80,7	70,7	64,2	78,0	37,7	54,0	42,5	47,4	53,6
	Central Bohemian Region	85,4	98,0	139,5	119,9	129,7	110,6	114,5	118,9	123,5	94,2	102,7	64,0	63,6	94,4
	South Bohemian Region	117,1	132,5	168,3	174,0	187,1	154,7	155,8	152,3	183,2	145,4	127,1	99,0	98,8	134,3
	Plzeň Region	99,8	113,6	142,7	183,4	120,0	141,7	133,9	106,9	130,5	109,5	103,8	64,0	63,3	96,0
	Karlovy Vary Region	96,9	68,1	89,4	109,9	86,8	93,2	90,5	71,5	83,8	77,8	71,9	43,6	45,4	65,3
	Ústí nad Labem region	94,8	61,0	74,6	71,9	69,1	58,2	71,6	60,9	72,6	70,9	54,9	34,9	42,2	55,8
	Liberec region	60,0	62,5	76,8	81,3	97,2	62,1	73,4	69,3	88,5	70,6	62,7	38,5	38,4	61,3
	Hradec Králové region	147,3	116,7	171,1	127,9	111,8	113,3	131,3	117,5	120,8	100,6	94,9	68,0	84,6	97,6
	Pardubice Region	138,1	174,1	204,0	162,5	147,3	124,7	158,5	151,0	193,0	120,8	140,3	97,0	93,7	132,2
	Vysočina region	131,7	137,5	192,2	170,0	160,5	155,3	157,8	172,9	174,1	126,5	140,5	93,0	88,2	132,5
	South Moravian Region	118,7	128,6	173,4	148,4	127,6	149,8	141,4	113,7	142,4	131,6	109,7	77,6	86,6	110,4
	Olomouc Region	85,1	80,4	120,9	111,2	107,7	94,9	99,9	123,9	132,2	108,4	102,6	83,5	75,5	104,2
	Zlín Region	119,3	96,3	114,7	89,3	82,0	89,4	98,5	96,6	140,6	101,3	94,9	96,5	92,8	103,6
	Moravian-Silesian Region	92,2	82,8	105,4	110,8	93,5	111,3	99,1	105,6	124,1	97,6	103,8	101,4	79,7	101,8
**Hospitalised (n=29,082)**		2463	2402	3130	3000	2664	2464	16123	2336	2773	2106	2165	1774	1805	12959
	% of total	23,5	23,4	23,0	23,6	22,5	21,1	22,9	20,6	20,8	20,3	21,5	23,1	23,4	21,4
**Sepsis (n=29,082)**		n.a.	n.a.	n.a.	n.a.	n.a.	n.a.	n.a.	60	82	69	47	64	64	386
	% of total	n.a.	n.a.	n.a.	n.a.	n.a.	n.a.	n.a.	0,5	0,6	0,7	0,5	0,8	0,8	0,6
**Imported cases (n=2,627)**		172	191	221	240	192	273	1289	288	392	46	73	240	299	1338
	% of total	1,6	1,9	1,6	1,9	1,6	2,3	1,8	2,5	2,9	0,4	0,7	3,1	3,9	2,2

Notes: [#] Since January 2018, surveillance has transitioned to the electronic reporting system (ISIN). [$] lower and upper quartiles (Q1, Q3), SD = standard deviation, [&] rest to 100 % were reported as probable cases, [^] rest to 100 % were reported as sporadic cases,

" for the first period notification rate is calculated towards population in 2014, for second period in 2020

Disclaimer: This supplementary material is hosted by Eurosurveillance as supporting information alongside the article "Epidemiology of human salmonellosis in Czechia — a country with the highest European notification rate, 2012 to 2023", on behalf of the authors, who remain responsible for the accuracy and appropriateness of the content. The same standards for ethics, copyright, attributions and permissions as for the article apply. Supplements are not edited by Eurosurveillance and the journal is not responsible for the maintenance of any links or email addresses provided therein.
